# Dendritic cells-derived interferon-λ1 ameliorated inflammatory bone destruction through inhibiting osteoclastogenesis

**DOI:** 10.1038/s41419-020-2612-z

**Published:** 2020-06-02

**Authors:** Yueqi Chen, Yiran Wang, Ruohui Tang, Jing Yang, Ce Dou, Yutong Dong, Dong Sun, Chengmin Zhang, Lincheng Zhang, Yong Tang, Qijie Dai, Fei Luo, Jianzhong Xu, Shiwu Dong

**Affiliations:** 1 Department of Orthopedics, Southwest Hospital, Third Military Medical University (Army Medical University), 400038 Chongqing, China; 2 Department of Biomedical Materials Science, Third Military Medical University (Army Medical University), 400038 Chongqing, China; 3 Department of Emergency, Daping Hospital, Third Military Medical University (Army Medical University), 400038 Chongqing, China; 4 State Key Laboratory of Trauma, Burns and Combined Injury, Third Military Medical University (Army Medical University), 400038 Chongqing, China

**Keywords:** Mechanisms of disease, Osteoimmunology

## Abstract

Bone infection contributing to inflammatory osteolysis is common in orthopedic surgery. The dynamic balance between bone formation and bone resorption is destroyed due to excessive osteoclast fusion and differentiation, which results in severe bone matrix loss. Many therapeutic approaches that restrain osteoclast formation and function act as efficient ways to prevent inflammatory bone erosion. We have demonstrated for the first time that dendritic cells-derived interferon-λ1 (IFN-λ1) inhibited inflammatory bone destruction in vivo and explored its underlying mechanisms on osteoclast formation in vitro. We found that IFN-λ1 was highly expressed in infectious bone tissue compared with that of non-infectious bone tissue. Additionally, dendritic cells marker genes such as CD80, CD86, and CD1a were higher expressed in infectious bone tissue than that of non-infectious bone tissue. Dendritic cells that were pretreated with LPS showed high expression of IFN-λ1. Moreover, conditioned medium of LPS-pretreated dendritic cells significantly inhibited osteoclast differentiation, as determined by TRAP staining assay. This suppressive effect was reversed by adding an IFN-λ1 monoclonal antibody. It was also investigated whether exogenous IFN-λ1 restrained osteoclastogenesis, bone resorption, F-actin ring formation, osteoclast-specific gene expression, release of pro-inflammatory cytokines, and translocation of p65 and NFATc1 by preventing the NF-κB signaling pathway and NLRP3 inflammasome formation, as well as by inducing the JAK-STAT signaling pathways in vitro. In vivo study indicated that IFN-λ1 prevents lipopolysaccharide (LPS)-induced inflammatory bone destruction by inhibiting excessive osteoclast fusion and bone resorption activity. In conclusion, our findings confirmed that dendritic cells-derived IFN-λ1 could attenuate osteoclast formation and bone resorptive activity in vitro and in vivo. These novel findings pave the way for the use of exogenous IFN-λ1 as a potential therapeutic treatment for excessive osteoclast-related diseases, such as inflammatory osteolysis, by regulating osteoclastogenesis to maintain the dynamic balance between bone formation and bone resorption.

## Introduction

Inflammatory diseases that occur in bone such as osteoarthritis (OA), rheumatoid arthritis (RA), orthopedic implant-associated infection, and osteomyelitis (OM) could contribute to focal erosion and an instant imbalance in dynamic bone matrix homeostasis^1–3^. OM often results in severe bone destruction, bone nonunion, and delayed fracture healing, which is severe challenges for orthopedic surgeon^4^. Moreover, some components are synthesized and released by pathogenic bacteria, which increase the risk of bone metabolic disorders^5,6^. OM also contributes to amputation and even death due to a lack of vascularization and excessive osteoclasts regulated bone resorption activity^7^. The pro-inflammatory cytokines in necrotic bone tissue induce the differentiation of osteoclasts^8^. Bone infection is rarely resolved without medical intervention and is difficult to cure due to the widespread antimicrobial resistance of *Staphylococcus aureus*, as well as the induction of bone damage that effectively limits antibiotic delivery and immune cell influx^9^. Therefore, there is also a clinical need to develop novel immunotherapies that target the excessive osteoclasts in the treatment of bone infectious diseases.

Bone represents a unique niche for invading bacterial pathogens as it is constantly undergoing turnover by bone-forming osteoblasts and bone-resorbing osteoclasts^10–12^. Osteoclasts (OCs), which are derived from the haematopoietic stem cells (HSCs), are formed through cell fusion, differentiation, and maturation and are stimulated by many pro-inflammatory cytokines^13^. As is known, macrophage colony-stimulating factor (M-CSF) and receptor activator of nuclear factor-κB ligand (RANKL) are the most essential cytokines for regulating osteoclastogenesis^14^. OCs make crucial role in maintaining the dynamic balance in the physiological bone microenvironment due to their specific effect on absorbing bone matrix^15^. Many studies have extensively documented that the interferon-α/β/γ system participated in the regulation of immune responses and bone metabolism, acting as a crucial bridge between immune system and bone metabolic system^16^. Interferon-α/β/γ could strongly inhibit RANKL-induced osteoclast differentiation, fusion and resorptive activity by blocking the RANKL-RANK signaling pathway and suppressing NF-κB signaling^17^.

Among the three types of interferon families, type III interferons also play vital role in regulating immune response and are composed of interferon lambda-1 (IFN-λ1), lambda-2 (IFN-λ2) and lambda-3 (IFN-λ3)^18^. Similar to the type-I interferon, type III interferons are induced by viral infections, as well as by a range of mitogens. Moreover, type III interferon have been shown to exhibit antiviral and immunoregulatory activities through the same signaling pathways as those utilized by type-I interferons^19^. The receptors of type III interferon include IFNLR1 and IL10R2. The discovery of the IFNLR and the similarity of its ligand to IFNα instigated the discovery of the IFNλs^20^. IFN-λ1 is the most potent IFN-λ molecule in humans, as well as the most abundant IFN-λ in serum, despite only existing as a pseudogene in mice. Previous studies on IFN-λ1 have focused on its function in some diseases, such as antiviral and anti-tumor effects, but fewer studies have focused on the association of IFN-λ1 with bone infections^21^. Owing to its efficiently immunoregulatory effect, we hypothesized that the negative regulation of IFN-λ1 on osteoclasts formation and function would be of great value for treating inflammatory osteolytic diseases.

## Results

### Traumatic osteomyelitis is associated with high-level IFN-λ1 level

**Fig. 1:**
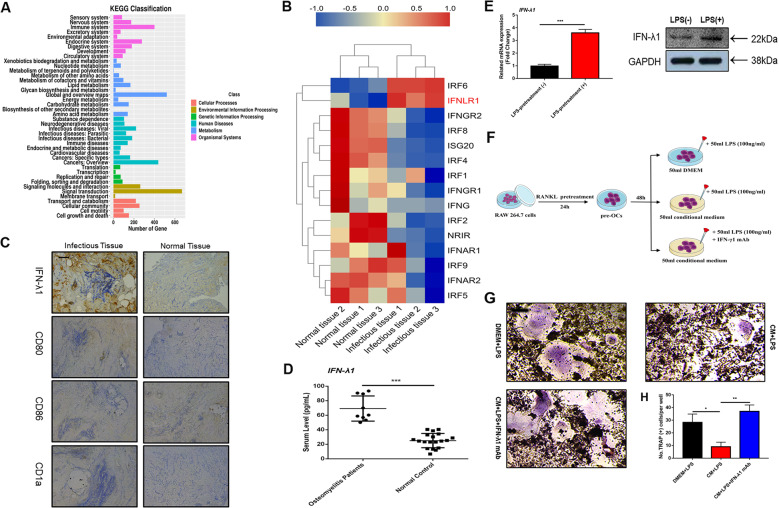
IFN-λ1 was higher expressed from dendritic cells during inflammatory statement. **a** The genes expression in immune system were analyzed by KEGG pathway enrichment analysis between chronic osteomyelitis-infected samples and non-infectious fractures samples. **b** Profiling of differentially expressed interferon-related genes between chronic osteomyelitis-infected samples and non-infectious fractures samples. **c** Representative images of immunohistochemical staining with monoclonal antibodies against IFN-λ1, CD80, CD86, and CD1a. **d** ELISA for IFN-λ1 concentrations from the serum of chronic osteomyelitis patients and non-infectious fracture patients. **e** Relative expression of IFN-λ1 on mRNA and protein level between LPS (100 ng/ml) pretreatment with dendritic cells and vehicle for 72 h. β-actin and GAPDH were used as an internal control. **f** Schematic representation of the experimental design of the establishment the co-culture between osteoclast and LPS-induced dendritic cells. **g** Representative images of TRAP staining of RAW264.7 cells treated with different groups. **h** Quantification of multinucleated TRAP-positive osteoclast number per well. The data in the figures represent the averages ± SD. Scale bars = 200 μm. Significant differences are indicated as *p < 0.05 or **p < 0.01 paired using Student’s *t*-test unless otherwise specified.

A previous study demonstrated that IFN-λ1 was presented at high levels in the serum of RA patients^22^. We also examined the serum level and messenger RNA (mRNA) or protein expression in human subjects with chronic osteomyelitis-infected and non-infectious closed fractures. Firstly, RNA-Seq analysis was utilized to examine the differences in expression between these two groups at the transcriptomic level. KEGG classification analysis showed that a high percentage of all genes were detected in the immune system (Fig. 1a). Interferon-λ1 related genes (such as IFNLR1) were highly expressed at the mRNA level (Fig. 1b). Interestingly, the blood samples in most osteomyelitis patients had high levels of IFN-λ1 compared with those of the non-infectious group (Fig. 1d). Additionally, immunohistochemical analysis of bone sections revealed significantly higher expression of IFN-λ1 in bone infectious tissue than that in non-infectious bone tissue (Fig. 1c). In order to make the confirmation that the derive of IFN-λ1, we have found that the expression of IFN-λ1 in dendritic cells (DCs) was also increased after stimulation with bacterial components such as LPS. In addition, we investigated that DC marker genes such as CD80, CD86 and CD1a were also expressed higher on infectious bone tissue than in non-infectious bone tissue, which were all the same area when we evaluation the expression of IFN-λ1. Moreover, the conditional medium (CM) of from DC2.4 cells that were pretreated with LPS for 72 h was collected. LPS pretreatment enhanced the mRNA and protein expression of IFN-λ1 compared with that of the control group (Fig. 1e). Then, 50% CM or Dulbecco’s modification of Eagle’s medium (DMEM) and 50% (100 ng/ml RANKL + 50 ng/ml M-CSF) were added to induce osteoclastogenesis, and the CM group had significantly inhibited osteoclast differentiation. Interestingly, administration of the monoclonal IFN-λ1 antibody could reverse the inhibitory effect (Fig. 1f). These results suggested that the high level of IFN-λ1 played an essential role in inflammatory bone destruction and dynamic bone matrix balance.

### IFN-λ1 prevented LPS-induced inflammatory bone destruction

**Fig. 2:**
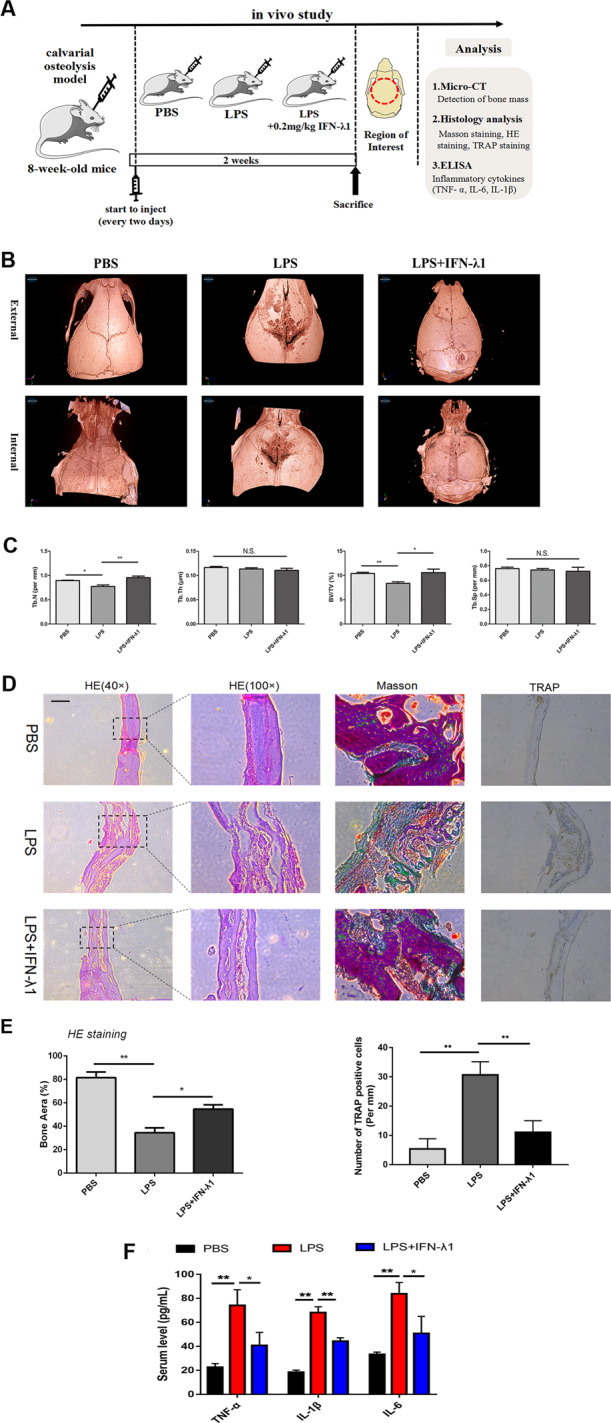
Exogenous IFN-λ1 inhibited LPS-induced inflammatory osteolysis. **a** Schematic representation of the design of in vivo experiments. **b** Representative 3D μCT images of reconstructed mouse calvarial (from internal to external/from external to internal). **c** Quantification of trabecular number (Tb. N), trabecular separation (Tb. Sp), trabecular thickness (Tb.Th), and trabecular bone volume fraction (BV/TV). **d** Representative images of cranial sections stained with H&E, Masson, and TRAP from each group. Scale bars=200μm. **e** Quantification of percentage of bone area in the border zone of the cranial H&E staining by using Image J software and the analysis of TRAP-positive cells in each group. **f** ELISA to detect the concentration of IL-1β, IL-6, and TNF-α in serum. The data in the figures represent the averages±SD. N.S. represented as no significant difference. Significant differences are indicated as *p<0.05 or **p<0.01 paired using Student’s *t-*test unless otherwise specified.

LPS could contribute to severe inflammatory osteolysis due to the production of pro-inflammatory cytokines^23^. A study has developed an efficient osteolysis mouse model^24^. After administration of LPS (10 mg/kg) in the absence or presence of IFN-λ1 (0.2 mg/kg) every 2 days for 28 days, the micro-CT and histological analysis were all performed (Fig. 2a). The µCT results showed that BV/TV and Tb.N were significantly inhibited by IFN-λ1 treatment (Fig. 2b, c). The area of bone loss was decreased not only from the internal but also from the external mouse skull. In addition, histological analysis also showed the same results. HE staining revealed that IFN-λ1 treatment obviously impeded the loss of bone area, which was consistent with the three-dimensional (3D) reconstruction images of the bone parameters (Fig. 2d, e). Furthermore, Masson staining showed that treatment with IFN-λ1 induced the formation of new bone tissue (Fig. 2d, e). Moreover, immunochemistry staining for TRAP has shown that treatment with IFN-λ1 strongly reduced the number of TRAP-positive cells (Fig. 2d, e). These results suggested that IFN-λ1 participated in negatively regulating inflammatory osteolysis in vivo.

### IFN-λ1 negatively regulated osteoclast formation and function without cytotoxicity

In order to detect the cytotoxicity of IFN-λ1, the CCK-8 results revealed that less than 200 ng/ml IFN-λ1 did not induce cytotoxicity or reduce the cell numbers (Fig. 2c, d). Interestingly, the total apoptosis rate was not influenced after adding different concentrations of IFN-λ1 by using flow cytometry (Fig. 2a, b). Thus, we chose 100 ng/ml IFN-λ1 to perform further experiments.

**Fig. 3:**
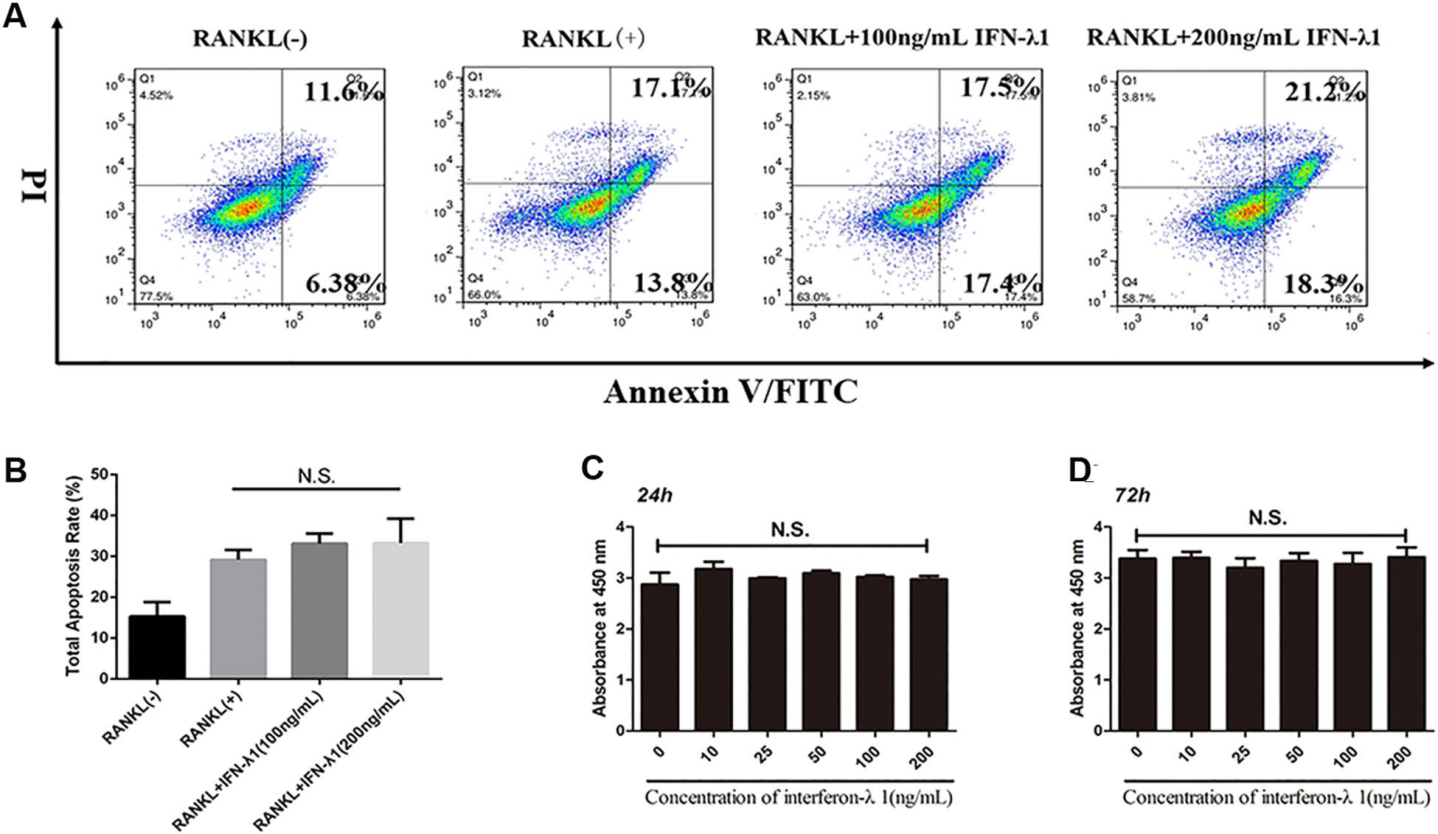
IFN-λ1 could not make cytotoxicity during osteoclastogenesis. **a** Flow cytometry analysis of the apoptosis rate of RAW264.7 cells treated with RANKL (100 ng/ml) and M-CSF (50 ng/ml) for 72 h with various doses of IFN-λ1. **b** Quantitative analysis of total apoptosis rate during osteoclastogenesis. **c**, **d** CCK-8 was performed in triplicate to analyze the cell viability of BMMs treated with varying doses of IFN-λ1 for 24 and 72 h with or without RANKL (100 ng/ ml) and M-CSF (50 ng/ml). The data in the figures represent the averages ± SD. N.S. represented as no significant difference. Significant differences are indicated as *p < 0.05 or **p < 0.01 paired using Student’s *t-*test unless otherwise specified.

To observe the effect of IFN-λ1 on RANKL-induced osteoclastogenesis, TRAP staining was performed. After incubation with RANKL in the presence or absence of 100 ng/ml IFN-λ1 for 72 h, we have found that 100 ng/ml IFN-λ1 significantly inhibited RANKL-induced osteoclast formation and the number of mature TRAP-positive (more than three nuclei) osteoclasts was also restrained (Fig. 3a, b). Notably, bone marrow macrophages (BMMs) were also incubated with 100 ng/ml IFN-λ1 for 5 days showed that IFN-λ1 also suppressed RANKL-induced osteoclast formation in BMMs from the TRAP staining (Fig. 3e, f). Moreover, FAK staining results showed that the number of mature osteoclasts and the average number of nucleus in multinucleated osteoclasts were significantly inhibited by treatment with IFN-λ1 (Fig. 5a, b). Taken together, we concluded that the inhibitory effect on RANKL-induced osteoclastogenesis was significantly mediated by the indicated dose of IFN-λ.

**Fig. 4:**
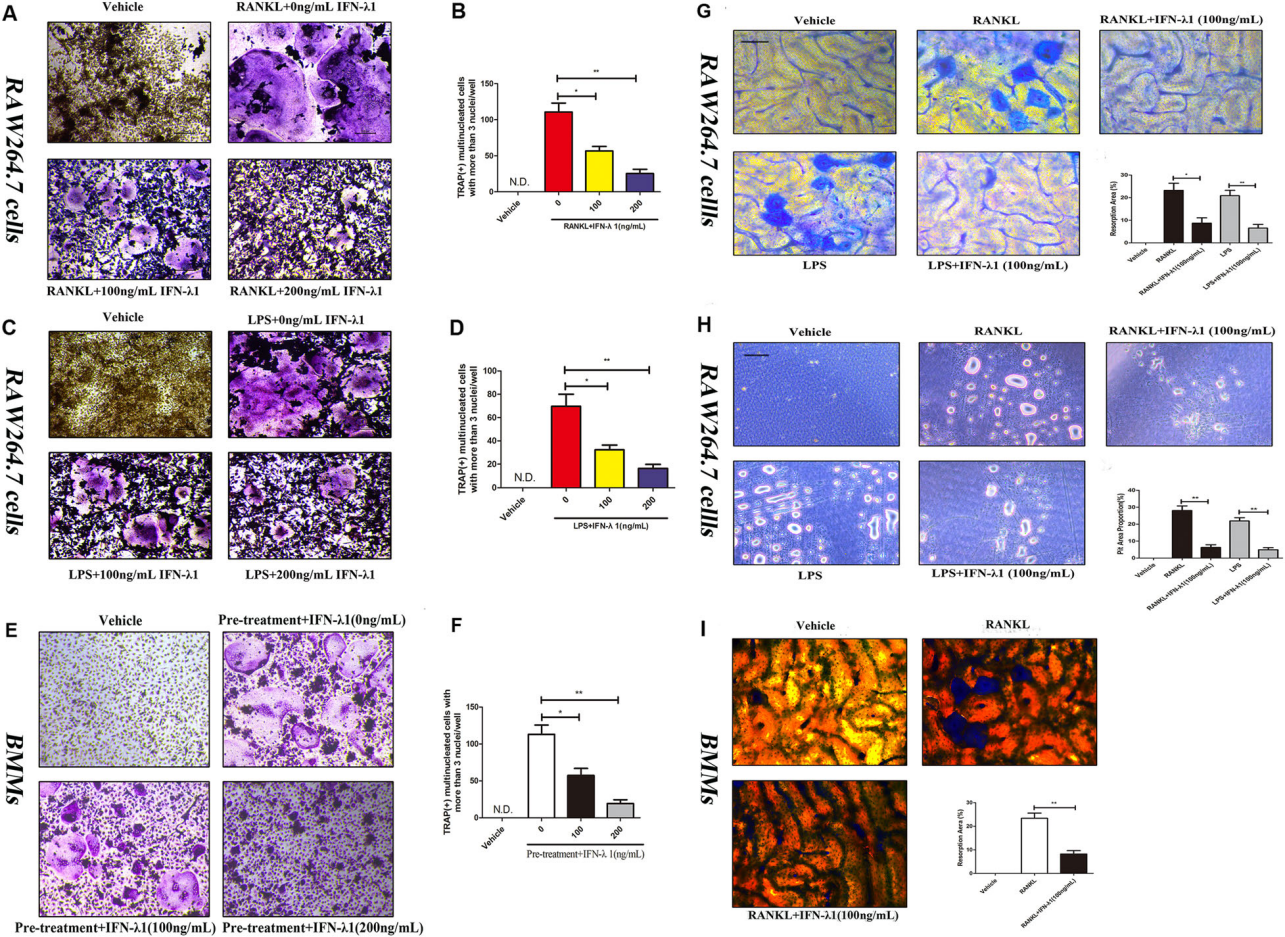
IFN-λ1 could inhibit osteoclast differentiation and bone resorption activity, respectively. **a**, **c**, **e** Representative TRAP stain images of RAW264.7 cells and BMMs treated with RANKL or LPS-induced osteoclastogenesis. **b**, **d**, **f** Quantification of osteoclasts number per well. **g** RAW264.7 cells were plated on the bone slices and were cultured with RANKL or LPS for 6 days in the presence or absence of 100 ng/ml IFN-λ1. Scale bar = 200 μm. Quantification of the bone resorption area on the bone slices. **h** RAW264.7 cells were plated on the Osteo Assay Surface and were cultured with RANKL or LPS for 6 days in the presence or absence of 100 ng/ml IFN-λ1. Scale bar = 200 μm. Quantification of the bone resorption area on the bone slices. **i** BMMs were plated on the bone slices and were cultured with RANKL for 6 days in the presence or absence of 100 ng/ml IFN-λ1. Scale bar = 200 μm. Quantification of the bone resorption area on the bone slices. The data in the figures represent the averages ± SD. N.S. represented as no significant difference. Significant differences are indicated as *p < 0.05 or **p < 0.01 paired using Student’s *t-*test unless otherwise specified.

**Fig. 5:**
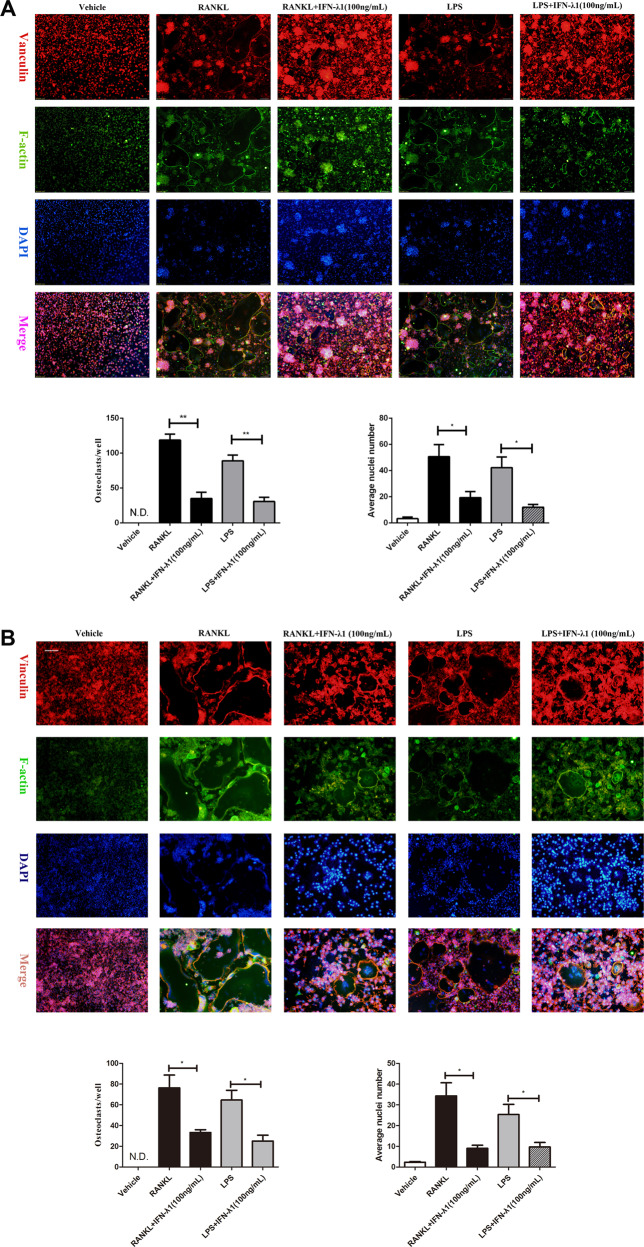
IFN-λ1 suppressed RANKL or LPS-induced osteoclast fusion significantly. **a** Representative images of FAK staining of RAW264.7 cells treated with RANKL and M-CSF alone or together with the indicated concentrations of IFN-λ1 treatment. F-actin using tetramethylrhodamine-conjugated phalloidin (red), focal contacts using anti-vinculin mAb, and nuclear counterstaining using DAPI (blue). Scale bar = 200 μm. Quantitative analysis of osteoclasts (nucleiå 3) and average osteoclast nuclei number in each field. **b** Representative images of FAK staining of RAW264.7 cells treated with RANKL and M-CSF alone or together with the indicated concentrations of IFN-λ1 treatment. F-actin using tetramethylrhodamine-conjugated phalloidin (red), focal contacts using anti-vinculin mAb, and nuclear counterstaining using DAPI (blue). Scale bar = 200 μm. Quantitative analysis of osteoclasts (nuclei > 3) and average osteoclast nuclei number in each field. The data in the figures represent the averages ± SD. Significant differences are indicated as *p < 0.05 or **p < 0.01 paired using Student’s *t-*test unless otherwise specified.

In order to detect a suitable concentration to complete LPS-induced osteoclastogenesis, we first investigated the cytotoxicity of different doses of LPS after incubation for 24 h and 72 h. We observed that less than 1000 ng/ml LPS did not change the proliferation of RAW264.7 cells. Thus, we chose 100 ng/ml LPS to induce osteoclast formation (Supplementary Fig. 1). LPS did not induce osteoclast formation without RANKL pretreatment. TRAP staining showed that pretreatment with RANKL for 24 h could significantly induce the formation of TRAP-positive cells and the number of osteoclasts compared to those of pretreatment with phosphate-buffered saline (PBS) for 24 h. In contrast, treatment with 100 ng/ml IFN-λ1 significantly reversed the effect of LPS-induced osteoclastogenesis (Fig. 4e, f). In addition, LPS-only treatment for 72 h did not induce TRAP-positive multinucleated cells (Supplementary Fig. 2). This illustrated that LPS-mediated osteoclastogenesis was not separable from RANKL pretreatment. Furthermore, the formation of the F-actin ring during LPS-induced osteoclastogenesis was also dramatically suppressed after treatment with 100 ng/ml IFN-λ1 (Fig. 5). Above these, it was suggested that IFN-λ1 could inhibit LPS-induced osteoclastogenesis.

Moreover, pit formation assay was performed to detect the effect of IFN-λ1 on osteoclast bone resorption activity. RAW264.7 cells were incubated with RANKL and LPS on bovine bone slides and osteo surface plates (Corning Osteo Assay Surface) for 5 days and the apparent bone degradation area was observed. Quantitative analysis showed that the resorption area was markedly altered by treatment with IFN-λ1 (Fig. 4g, h). Similarly, BMMs were incubated with RANKL onto bovine bone slides after treatment with or without IFN-λ1 for 7 days, and the bone resorption area was dramatically inhibited by IFN-λ1 (Fig. 4i). Thus, IFN-λ1 negatively regulated osteoclast bone resorption activity in vitro.

### IFN-λ1 suppressed the expression of osteoclast formation and function-related genes

**Fig. 6:**
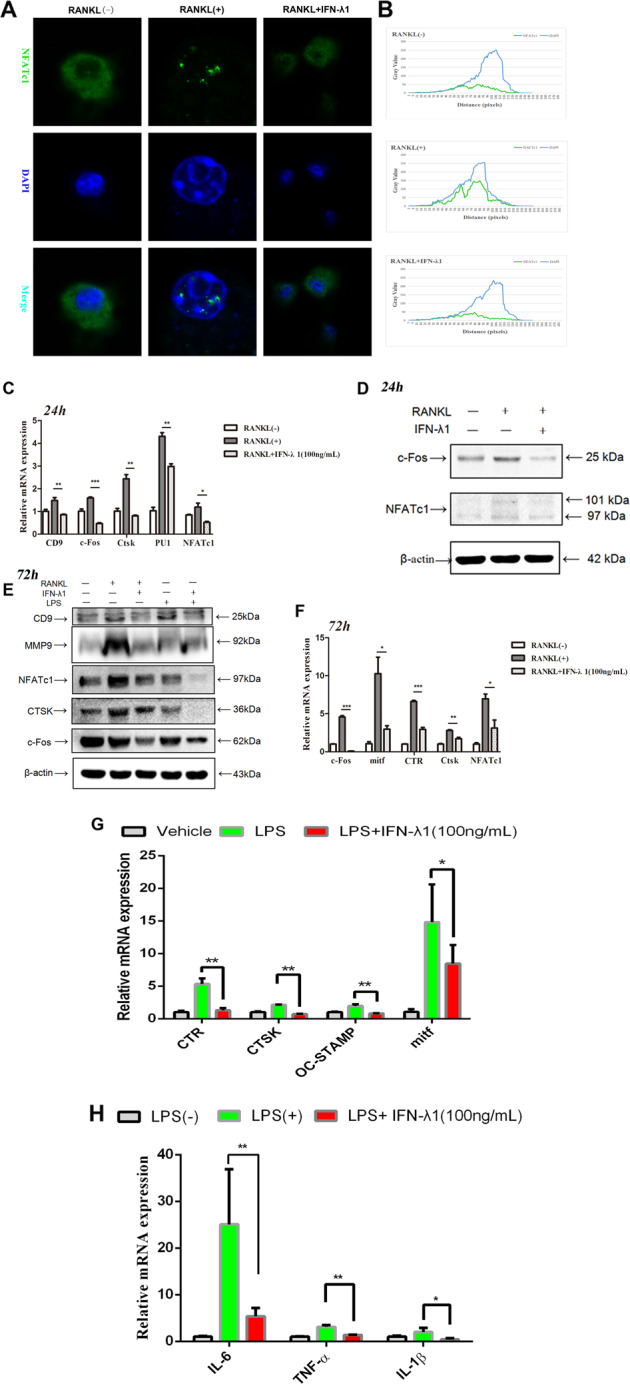
IFN-λ1 inhibited the nuclei translocation of NFATc1 and the expression of osteoclast-specific genes. **a** RAW264.7 cells were seeded in 96-well plates and treated with IFN-λ1 (100 ng/ml) for 24 h, followed by stimulation with 100 ng/ml RANKL and 50 ng/ml M-CSF. The intracellular location of the NFATc1 was observed by immunofluorescence staining using confocal microscopy. Scale bar = 800 μm. **b** The gray values of the Green and Blue staining were measured using the Image J software, and the mean values were plotted using excel. **c** Relative mRNA expression of CD9, c-Fos, Ctsk, PU.1, and NFATc1 during treatment with RANKL in the presence or absence of IFN-λ1 (100 ng/ml) for 24 h. **d** Relative expression of c-Fos and NFATc1 during treatment with RANKL in the presence or absence of IFN-λ1 (100 ng/ml) for 24 h in protein level. β-actin was used as an internal control. **e** Relative expression of CD9, MMP-9, CTSK, c-Fos, and NFATc1 during treatment with RANKL or LPS in the presence or absence of IFN-λ1 (100 ng/ml) for 72 h in protein level. β-actin was used as an internal control. **f** Relative mRNA expression of mitf, c-Fos, Ctsk, CTR, and NFATc1 during treatment with RANKL in the presence or absence of IFN-λ1 (100 ng/ml) for 72 h. **g** Relative mRNA expression of mitf, Ctsk, CTR, and OC-STAMP during treatment with LPS in the presence or absence of IFN-λ1 (100 ng/ml) for 72 h. **h** Relative mRNA expression of IL-1β, IL-6, and TNF-α during LPS-induced osteoclastogenesis in the presence or absence of IFN-λ1 (100 ng/ml). The data in the figures represent the averages ± SD. Significant differences are indicated as *p < 0.05 or **p < 0.01 paired using Student’s *t-*test unless otherwise specified.

Previous studies have shown that RANKL stimulation alone induced the translocation of NFATc1 from the cytoplasm to the nucleus to regulate the expression of many OC-related marker genes during osteoclastogenesis^25^. To detect the effect of IFN-λ1 on NFATc1 translocation, RAW264.7 cells were incubated with RANKL in the presence and absence of IFN-λ1 for 24 h, and we observed that the average fluorescence density was significantly suppressed after the treatment with IFN-λ1 compared to that triggered only with RANKL (Fig. 6a, b). To investigate the differentiation of different stages of osteoclasts, RAW264.7 cells were incubated with RANKL for 24 h to obtain preosteoclasts or 72 h to mature osteoclasts. The results illustrated that treatment with IFN-λ1 could significantly inhibit both the fusion of monocytes to preosteoclasts and mature multinucleated osteoclasts. The expression of c-Fos and NFATc1 were also restrained both at the mRNA and protein levels. (Fig. 6e, f). Moreover, the mRNA expression of early stage genes that regulated the formation of OCPs such as CD9, PU1 and Ctsk was attenuated (Fig. 6c). Other genes such as CTR, Ctsk and mitf that regulate mature osteoclast differentiation and bone resorption activity were also ameliorated in the presence of IFN-λ1 at the mRNA level (Fig. 6f). MMP9 and Ctsk were reported to participate in eroding the bone matrix by mature osteoclasts^26^. Treatment with IFN-λ1 dramatically inhibited the expression of these genes at the protein level, which revealed that the bone erosion activity of excessive osteoclasts was improved by IFN-λ1 (Fig. 6e). CD9 acts as a membrane protein that regulates the fusion of osteoclasts derived from monocytes^27^. IFN-λ1 also similarly inhibited the expression of CD9 at the protein level (Fig. 6e). Based on these results, IFN-λ1 regulated the genes expression during the different stages of osteoclast differentiation induced by both RANKL and LPS (Fig. 6g).

### IFN-λ1 inhibited the production of pro-inflammatory cytokines and the formation of NLRP3 inflammasome during osteoclastogenesis

The induction of releasing inflammatory cytokines and NLRP3 inflammasome formation were acted as promoter during osteoclastogenesis^28^. Owing to the suppressive effect of IFN-λ1 on LPS-induced osteoclastogenesis, the production of pro-inflammatory cytokines such as IL-1β, IL-6, and TNF-α after treatment with LPS and IFN-λ1 should also be investigated. During RANKL pretreatment for 24 h, LPS was added for another 48 h to induce mature osteoclast differentiation, and large amounts of IL-1β, IL-6, and TNF-α were also produced. Real-time qPCR results revealed that IFN-λ1 significantly inhibited the expression of these genes during LPS-induced osteoclastogenesis (Fig. 6h). We also performed ELISA to detect the concentration of these pro-inflammatory cytokines in the serum during LPS-mediated inflammatory osteolysis in vivo (Fig. 2h).

**Fig. 7:**
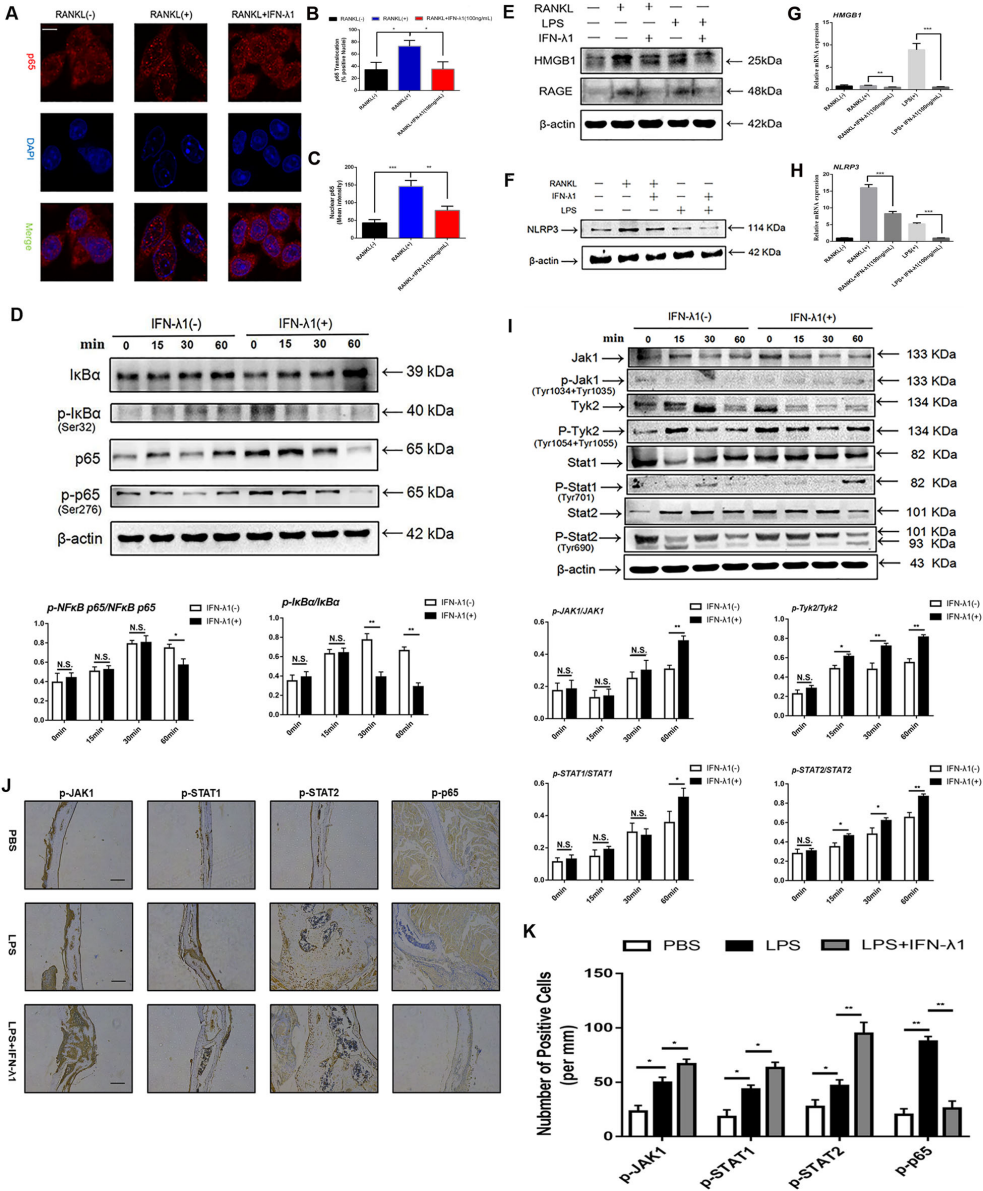
IFN-λ1 inhibited the classical NF-κB signal pathway, the formation of NLRP3 inflammasome and activated JAK-Stat signal pathway. **a** RAW264.7 cells were seeded in 96-well plates and treated with IFN-λ1 (100 ng/ml) for 60 min, followed by stimulation with 100 ng/ml RANKL and 50 ng/ml M-CSF. The intracellular location of the NF-κB p65 was observed by immunofluorescence staining using confocal microscopy. Scale bar = 800 μm. **b** Quantitative analysis of the percentage of positive cells (NF-ĸB p65 translocation from cytosol to nuclear) in all cells. **c** Quantitative analysis of the mean intensity of NF-ĸB p65 in the cells nuclear. **d** RAW264.7 cells were stimulated with RANKL with or without IFN-λ1 (100 ng/ml) for the 0–60 min. The cell lysates were analyzed using western blotting for p-NFκB p65, NF-κB p65, p-IκBα, and IκBα. β-actin was used as an internal control. **e**, **f** The expression of HMGB1, RAGE, and NLRP3 during osteoclastogenesis in the presence or absence of IFN-λ1 (100 ng/ml) on protein level. β-actin was used as an internal control. **g**, **h** Relative expression of HMGB1 and NLRP3 during osteoclastogenesis in the presence or absence of IFN-λ1 (100 ng/ml) on mRNA level. **i** RAW264.7 cells were stimulated with RANKL with or without IFN-λ1 (100 ng/ml) for the 0–60 min. The cell lysates were analyzed using western blotting for p-Jak1, Jak1, p-Tyk2, Tyk2, p-Stat1, Stat1, p-Stat2, and Stat2. β-actin was used as an internal control. **j** Representative images of immunohistochemical staining with monoclonal antibodies against p-p65, p-JAK1, p-STAT1, and p-STAT2. Scale bar = 200 μm. **k** Quantitative analysis of p-p65, p-JAK1, p-STAT1, and p-STAT2-positive cells per mm in each field. The data in the figures represent the averages ± SD. Significant differences are indicated as *p < 0.05 or **p < 0.01 paired using Student’s *t-*test unless otherwise specified.

Additionally, the restricted activation of the NLRP3 inflammasome during inflammatory osteolysis was an efficient mechanism to regulate the formation of mature OCs and the release of inflammatory cytokines. During RANKL and LPS-induced osteoclastogenesis, the NLRP3 inflammasome was reported to be highly induced^29^. After stimulation with many inflammatory stimuli (such as DAMPs and their production), the release of IL-1β was also necessary to regulate inflammatory reactions, which was crucially regulated by the synthesis of NLRP3 inflammasome^30^. In vivo analysis showed that treatment with IFN-λ1 decreased the level of IL-1β in the serum. To determine the inhibitory effect on the release of IL-1β, we measured the expression of NLRP3 at the protein level. As expected, NLRP3 was significantly suppressed during RANKL and LPS-induced osteoclast fusion and differentiation (Fig. 7g). Many studies have noted that activation of NLRP3 inflammasome facilitated HMGB1 translocation from the nucleus to the cytoplasm^31^. Previous studies showed that extracellular HMGB1 binding to RAGE regulated the organization of actin cytoskeleton and participated in osteoclastogenesis^32^. To investigate whether IFN-λ1 affected NLRP3 inflammasome formation in an HMGB1-dependent manner during osteoclastogenesis, we performed qPCR and western blotting to examine the expression of HMGB1, RAGE, and NLRP3 at mRNA and protein levels. HMGB1 release, as well as extracellular HMGB1, in RANKL- or LPS-induced osteoclastogenesis and osteoclast activation were impaired in the presence of IFN-λ1. Consistently, the expression of its receptor, RAGE, was also attenuated. Moreover, the NLRP3 inflammasome was dramatically induced only when stimulated with RANKL and LPS during osteoclastogenesis. Conversely, treatment with IFN-λ1 reversed this activation (Fig. 7f–i). Taken together, these results revealed that IFN-λ1 suppressed the activation of NLRP3 inflammasome via HMGB1-RAGE-dependent manner and the release of pro-inflammatory cytokines due to its anti-inflammatory effect.

### IFN-λ1 restrained RANKL-induced osteoclast differentiation and function via NF-κB signal pathway

Owing to the inhibitory effects on the release of pro-inflammatory cytokines and the formation of NLRP3 inflammasome, which contributed to the suppression of osteoclastic genes and potential mechanisms. NF-κB signal pathway was regarded as play crucial role in regulating osteoclast differentiation and bone resorption due to its regulatory role in inflammation^33^. Additionally, many associated genes that plays vital roles in osteoclastogenesis were regulated by this signaling pathway. To detect whether IFN-λ1 attenuated osteoclastogenesis by blocking NF-κB signaling to inhibit c-Fos-NFATc1 signaling pathway after RANKL stimulation, we utilized western blotting and p65 immunofluorescence. Many studies have revealed that NF-κB p65 protein translocation from the cytoplasm to the nucleus in response to different stimuli^34,35^. During osteoclast fusion and differentiation, NF-κB p65 was rapidly transported from the cytoplasm to the nucleus after RANKL stimulation^36^. Thus, we examined the location of NF-κB p65 during RANKL stimulation in the presence or absence of IFN-λ1. Most of p65 protein located in the cell nucleus after incubation with RANKL alone. However, administration of IFN-λ1 significantly reversed this nuclear translocation (Fig. 7a–c). Similarly, we also found that the phosphorylation of NF-κB p65 was inhibited after treatment with IFN-λ1 compared that of RANKL stimulation alone. The phosphorylation, degradation of IκB-α are crucial for dissociating the p65 subunit and translocating it from the cytoplasm to the nucleus to initiate the expression of osteoclast marker genes after treatment with RANKL^36^. We determined that IFN-λ1 inhibited the expression of IκB-α and p-IκB-α at the protein level (Fig. 7d). Interestingly, we also found that the expression of p-p65 was inhibited after treatment with IFN-λ1 in vivo (Fig. 7j, k). Collectively, we concluded that IFN-λ1 made an inhibitory role in regulating activation of the NF-κB signaling pathway during RANKL stimulation.

### Restraining NF-κB in osteoclastogenesis via activating JAK-STAT signal pathway dependently after stimulation with IFN-λ1

NF-κB-dependent activation of suppressor of cytokine signaling-3 (SOCS-3) protein expression negatively affected signal transducer and activator of transcription-1 (STAT)-1 and STAT-2 phosphorylation during type-I IFN responses^37^. Moreover, the Janus kinase (JAK)-STAT signaling pathway was crucial for regulating osteoclast differentiation and bone resorption activity^38^. JAK proteins can activate immune cells, induce pro-inflammatory cytokine expression and transmit cytokine signaling^39^. RAW264.7 cells were incubated with RANKL to stimulate signal transduction. Following stimulation with RANKL alone or in combination with 100 ng/ml IFN-λ1 for 60 min, the phosphorylation of JAK1 (at Tyr 1034 + Tyr 1035), Tyk2 (at Tyr 1054 + Tyr 1055), STAT1 (at Tyr 701), and STAT2 (at Tyr 690) was induced after treatment with IFN-λ1. The total expression of JAK1, Tyk2, STAT1 and STAT2 were showed that no significant difference in response to IFN-λ1 treatment (Fig. 7j). Additionally, we also found that the expression of p-JAK1, p-STAT1, and p-STAT2 was induced significantly after treatment with IFN-λ1 in vivo (Fig. 7j, k). Collectively, these results illustrated that IFN-λ1 acted as a promoter to activate JAK1-STAT1/STAT2 signaling pathway.

## Discussion

Inflammatory osteolysis is a kind of bone disease that is caused by excessive osteoclast bone resorption activity, resulting in low bone mass and leading to a high risk of fracture and difficult bone regeneration. The dynamic balance between osteoblast-mediated bone formation and osteoclast-mediated bone resorption was destroyed due to the inflammatory microenvironment, which was regulated by the release of many inflammatory cytokines (such as IL-6, IL-1β, TNF-α, IL-17, et al.), resulting in the promotion of the bone erosion activity of osteoclasts. Currently, there was a problem in developing an effective way to treat bone infection due to the clinically activated antibiotic phenomenon. Therefore, regulating osteoclastogenesis and controlling the activity of osteoclasts were necessary to establish novel treatment strategies to cure inflammatory osteolysis. As the link between the immune and skeletal systems during bone infection has attracted increasing attention, we further investigated the role of IFN-λ1 in regulating osteoclast differentiation and its value in treating inflammatory osteolysis.

Previous studies found that *Staphylococcus aureus* was the most significant pathogen that contributed to excessive bone erosion. Not only the components of this bacteria but also the production of many pro-inflammatory cytokines promote osteoclast activity, causing an imbalance in the dynamic bone matrix. Bone infection stimulated many immune cells that participate in the process of bone regeneration^40^. DCs could also secret various chemokines and interleukins to attract other immune cells to complete bone regeneration and act as crucial antigen-presenting cells to initiate immune reaction during bone infection^41^. Secreted cytokines could be divided into two types based on their different effects on osteoclastogenesis. One type includes the IL-1 family, such as IL-6, which could significantly induce osteoclast fusion and differentiation to activate bone resorption activity. Another type includes the IL-10 family and interferon-related cytokines. In contrast, they played suppressive role in regulating osteoclastogenesis in excessive osteoclast-related bone diseases^42^.

Many studies have illustrated that IFN-λ1 could be highly secreted by DCs during many inflammatory diseases such as asthma^43^. IFN-λ1 was a unique participant in the IFN family and was regarded as type III IFN (IFN-λ1, IFN-λ2, and IFN-λ3). As immune cells both produce and respond to IFN-λ, they were likely to play an important role in the immune interface. Bone infection could result in the activation of the immune system, and many immune cells and cytokines that were released by immune activation are also changed. In clinical samples, IFN-λ-related ligands in infectious tissue were higher than those in connected non-infectious tissue, as determined by using transcriptome sequencing. Furthermore, the concentration of IFN-λ1 in the serum of bone infection patients was high. Additionally, there were more IFN-λ1-positive cells in the infectious tissue than in the control groups. Owing to the increased expression of IFN-λ1 during bone infection, we hypothesized that IFN-λ1 was necessary in treating this inflammatory bone disease. Previous studies have found that DCs were associated with many types of T cells, acting as antigen-presenting cells (APCs) during infections^44^. Additionally, it was reported that immature DCs could transdifferentiate to mature OCs faster than those fused from monocytes^42^. In periodontitis, the bacterial components in the environment of DCs contribute to DC trans-differentiation to mature OCs^45^. The expression of IFN-λ1 in DCs was also increased after stimulation with bacterial components such as LPS^46^. Therefore, IFN-λ1 might participate in the fusion and differentiation of osteoclasts derived from monocytes. Because of this finding, we examined the direct and indirect effects of IFN-λ1 on osteoclast differentiation. Firstly, we investigated whether treatment with IFN-λ1 significantly inhibited OC formation and bone resorption activity during RANKL or LPS induction in vitro. Injection of IFN-λ1 also protected against bone loss in calvarial osteolysis model in vivo. Moreover, the indirect effect of IFN-λ1 on osteoclastogenesis was also detected. We demonstrated that treatment with IFN-λ1 contributed to the production and release of pro-inflammatory cytokines such as IL-6, IL-1β, and TNF-α. In addition, the release of HMGB1 from the nucleus to the cytoplasm and the formation of NLRP3 inflammasome were induced after only treatment with LPS, whereas IFN-λ1 reversed these effects. These results suggested that IFN-λ1 directly inhibited both RANKL and LPS-induced osteoclast formation and bone resorption and IFN-λ1 indirectly controlled the release of pro-inflammatory cytokines (IL-6, IL-1β, and TNF-α).

NFATc1 acted as a master gene, which was regulated by classical NF-κB, JAK-STAT, c-Fos transcriptional factors. In order to maintain bone homeostasis, NFATc1 could regulate many osteoclast marker genes, such as Ctsk, MMP-9, CD9, and others. Treatment with IFN-λ1 has significantly downregulated the expression of Ctsk, MMP-9, CD9, PU1, and mitf during osteoclastogenesis at different levels. The NF-κB signaling pathway participates in regulating osteoclastogenesis in a direct or indirect way by influencing inflammatory cytokines. In the classical NF-κB signaling pathway, NF-κB p65 was inactive when it was in the cytoplasm, whereas when it was translocated into the nucleus, it was activated and triggered the formation of an NF-κB-related complex. The phosphorylation of IκB-α also activated the NF-κB signaling pathway through ubiquitination and subsequent degradation of the IKK complex. We found that RANKL stimulated the translocation of NF-κB p65 protein from the cytoplasm to the nucleus, and this effect was reversed by treatment with IFN-λ1. Additionally, the phosphorylation of the IκB-α and NF-κB p65 was also significantly inhibited by IFN-λ1. Currently, HMGB1 exhibited pro-inflammatory cytokine-like activity and participated in the activation of the innate immune response in pathological processes in the extracellular space. HMGB1 was also an inducer in RANKL-induced osteoclastogenesis after binding to its receptors such as RAGE and TLR2/4/9^47^. We found that IFN-λ1 controlled the binding of HMGB1 and RAGE during RANKL and LPS-induced osteoclastogenesis, which suggested that the release of HMGB1 from the nucleus to the cytoplasm to promote osteoclast formation was inhibited in a RAGE-dependent manner. What’s more, the NLRP3 inflammasome also regulated bone homeostasis, acting not only in the context of severe inflammation, but also in estrogen deficiency and other crucial elements. Previous studies have found that activation of the NLRP3 inflammasome in the OC lineage resulted in severe bone resorption in the absence of inflammation. Many studies have shown that NF-κB signaling pathway played an essential role in the formation of NLRP3 inflammasome. We also found that IFN-λ1 acted as an inhibitor in regulating the NF-κB signaling pathway to exert an anti-inflammatory effect. Interestingly, we further investigated whether NLRP3 was also suppressed by IFN-λ1, which was consistent with the inhibitory effect on the NF-κB signaling pathway.

Inhibitory effects related to osteoclastogenesis may be regulated through the interferon stimulated gene factor 3 (ISGF3) complex, which is composed of signal transducers and activators of transcription-1 (STAT1) and STAT2 and interferon regulatory factor 9 (IRF9)^48^. ISGF3 binds with the interferon stimulated response element (ISRE) to initiate subsequent gene mediated transcription events such as the classical c-Fos-NFATc1 signal pathway^49^. Similar with type-I interferon triggering the formation of the ISGF3 complex, IFN-λ1 also induced the JAK1-Tyk2-STAT cascades and inhibited NF-κB translocation from the cytoplasm to the nucleus and the expression of osteoclast-specific genes.

**Fig. 8:**
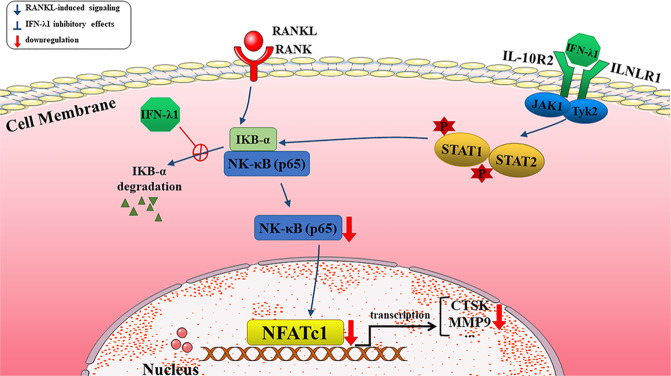
Schematic diagrams showing the potential mechanism in the protective effects of dendritic cells derived IFN-λ1 on inflammatory bone destruction through inhibiting osteoclastogenesis.

In summary, IFN-λ1 was more highly expressed in infectious tissue than in normal bone tissue, which contributed to activating inflammatory reactions. Moreover, the calvarial osteolysis model showed that treatment with IFN-λ1 dramatically prevented bone loss by regulating the inflammatory microenvironment. In addition, exogenous IFN-λ1 reversed the pathogenic process by regulating the release of pro-inflammatory cytokines. IFN-λ1 played an inhibitory role in osteoclast formation and function by directly inducing JAK-STAT signaling pathway. Generally, IFN-λ1 dramatically stimulated the JAK-STAT signaling pathway to affect NF-κB translocation, thereby dramatically inhibiting the release of pro-inflammatory cytokines and the formation of the NLRP3 inflammasome (Fig. 8). As a result, IFN-λ1 served as a novel therapeutic target for the treatment of excessive osteoclast bone infectious diseases.

## Materials and methods

### Cells, media, and reagents

Recombinant Human IFN-λ1 Protein (1598-IL-025/CF), recombinant murine TRANCE/RANKL (462-TEC-010/CF) and recombinant murine M-CSF (416-ML-010) were from R&D Systems (Minneapolis, USA). Antibodies against c-FOS, NFATc1 and MMP-9 were from Cell Signaling Technology Inc (Danvers, USA). Antibody against JAK1, p-JAK1, Tyk2, p-Tyk2, STAT1, p-STAT1, STAT2, p-STAT2, TRAP, CD9, CTSK, NLRP3, HMGB1, and RAGE were purchased from bioss (Beijing, China). Antibody against IFN-λ1(IL-29), CD80, CD86 and CD1a was obtained from Abcam (Cambridge, USA). Antibodies against p65, p-p65, IκBα, p-IκBα, and β-actin were from Bioworld Technology (St. Louis Park, MN, USA). DMEM, α-minimal essential medium (a-MEM), and fetal bovine serum (FBS) were purchased from Thermo Fisher Scientific (Waltham, MA, USA). Mouse IL-1β, IL-6, and TNF-α ELISA Kit were obtained from Dakewe Biotech co. Ltd (Shenzhen, China). Human IFN-λ1 ELISA Kit was obtained from Bioss (Beijing, China). Osteo Assay Surface was purchased from Corning (Corning, USA). CCK-8 kit was purchased from Dojindo Molecular Technologies (Tokyo, Japan). The TRAP stain kit and Lipopolysaccharides from Escherichia coli O55:B5 were from Sigma-Aldrich (St. Louis, USA). The Focal Adhesion and Actin Cytoskeleton Staining Kit were obtained from Millipore (Darmstadt, Germany). RAW264.7 cells (mouse macrophage cells) and BMMs were obtained from the American Type Culture Collection (Rockville, MD, USA). DC2.4 cells (mouse bone marrow-derived dendritic cells) were obtained from the Jinmai biotechnology (Chongqing, China). RAW264.7 cells and bone marrow monocytes (BMMs) were cultured with condition medium (CM) of DC2.4 cell lines with LPS (100 ng/ml) for 3 days. To block IFN-λ1 in the CM of DC2.4 cells, we used monoclonal antibodies against IFN-λ1 (Abcam) in a concentration of 50 μg/ml, and cultured for another 96 h.

### Human clinical samples

Three patients diagnosed with chronic osteomyelitis and three patients with non-infectious closed fractures were selected in this study. Clinical specimens were collected from Army medical university Southwest Hospital (Chongqing, China). All samples with the size of 1 × 1 cm, which were obtained from the fracture site during surgery. The study was allowed the law and approved by Southwest Hospital Clinical Ethics Committee.

For histological analysis, the samples were fixed in 4% paraformaldehyde (PFA) for 24 h and then decalcified by 10% EDTA solution for one month, and the solution was changed every three days. Samples were prepared into 5 μm thickness bone slices after paraffin embedded. Standard immunohistochemistry staining for IFN-λ1 was performed using a primary antibody against IFN-λ1, CD80, CD86, CD1a (1:100) on sections. Stained sections were observed by microscopy (Leica, DMI 6000B, Germany).

For measurement the serum level of IFN-λ1 among clinical patients and healthy control, blood samples were collected. The serum of human patients and healthy controls were obtained from whole blood after standing for 30 min at room temperature and centrifugation at 3000 × *g* for 10 min at 4 °C. Levels of IFN-λ1 in serum were measured with mouse ELISA kits according to the manufacturer’s instruction respectively.

### Transcriptomic sequencing analyses

The clinical bone tissue was collected and stored at –80 °C. In order to measure the expression of genes on mRNA level, the total RNA of these bone tissue was extracted. RNA sequence was performed by Sinotech Genomics Co., Ltd. Total amount of 2 µg RNA per sample was used as input material for the RNA sample preparations. RNA-seq libraries were prepared using NEBNext® UltraTM RNA Library Prep Kit for Illumina® (NEB, USA) following the manufacturer’s recommendations and index codes were added to attribute sequences to each sample. The log1.5 was transformed, further calculated and displayed as heatmaps to help visualize differential expression. Through screening comparison, H-cluster analysis was used to analyze the expression of differential genes and functional enrichment was studied by GO and KEGG analysis.

### Cell isolation and culture

RAW264.7 cells were cultured in DMEM complete medium supplemented with 1% penicillin–streptomycin (Thermo Scientific, USA) and 10% FBS at 37 °C in 5% CO2 condition. Bone marrow monocytes (BMMs) were obtained from 8-week-old C57/BL6 mice. The whole procedure was performed according to the guidelines of animal care and use committee of Army Medical University. Briefly, the cells were isolated from the femur and tibia bone marrow and cultured for 3 days in α-MEM supplemented with 10% FBS and 30 ng/ml M-CSF. The attached cells were considered as BMMs.

### Cytotoxicity and cell apoptosis assays

The cell proliferation and viability of RAW264.7 cells were evaluated by Cell Counting Kit (CCK)-8 assays (Dojindo Molecular Technologies, Tokyo, Japan). RAW264.7 cells (3 × 10^3^ cells/well) were seeded onto 96-well plates at 2 × 10^3^cells/well for 24 h and 72 h after treating with different concentrations of IFN-λ1. A 10% CCK-8 solution was added into each well, and the plates were incubated at 37 °C in a 5% CO_2_ atmosphere for 2 h. The absorbance at 450 nm was detected on the instrument (BioTek, Synergy H4, USA).

For detecting the effect of IFN-λ1 on cell apoptosis, we have used flow cytometry (BD, Triangle, NC, USA). The RAW264.7 cells were seeded onto 6-wells plate, they were incubated and divided into 50 ng/ml M-CSF group, 100 ng/ml RANKL + 50 ng/ml M-CSF group, 100 ng/ml RANKL + 50 ng/ml M-CSF group + 100 ng/ml IFN-λ1 group, and 100 ng/ml RANKL + 50 ng/ml M-CSF group + 200 ng/ml IFN-λ1 group. These groups were incubated for 48 h. The Annexin V-FITC/PI staining was performed to detect apoptosis of cultured cells. The process is consistent with the instructions (Life Technologies, USA).

### In vitro assays for osteoclast differentiation, fusion, and function

For tartrate-resistant acid phosphatase (TRAP) staining assay, cells were fixed with 4% paraformaldehyde for 15 minutes after being washed twice with PBS and then stained with TRAP staining solution according to the manufacturer’s directions. TRAP-positive multinucleated cells containing three or more nuclei were identified as osteoclasts and counted under the optical microscope (DMI 6000B; Leica Microsystems, Wetzlar, Germany). In addition, RAW264.7 cells were seeded into 96-well plates at a density of 3 × 10^3^cells/well and pretreated with PBS or RANKL (100 ng/ml) and M-CSF (50 ng/ml) for 24 h. Successively, the medium was replaced with fresh medium containing PBS or LPS (100 ng/ml) alone or together with IFN-λ1 for additional 48 h to generate multinucleated osteoclasts. After the culture, cells were performed for TRAP staining. TRAP-positive multinucleated cells containing three or more nuclei were counted. The purpose was to investigate the effects of IFN-λ1 on osteoclastogenesis under LPS-induced inflammatory conditions.

For focal adhesion and actin cytoskeleton staining, cells were fixed with 4% paraformaldehyde for 20 min and permeabilized with 0.1% Triton X-100 for 5 min at room temperature. After being blocked with the blocking buffer for 30 min, cells were stained with anti-vinculin antibody (1:300) and TRITC conjugated Phalloidin (1:500) revealed with Alexa Fluor 488-conjugated secondary antibody. Finally, nuclei staining was performed by DAPI (1:1000) for 5 min. Fluorescence microscopy was conducted to observe the cells.

For pit formation assay, RAW264.7 cells were incubated in 96-well plates covered with bovine bone slices (Jelling, Denmark) of 1 × 10^4^ cells/well. Cells were incubated with RANKL (100 ng/ml) and M-CSF (50 ng/ml) for 5 days. Bleach solution was added to 96-well osteo surface plates to remove cells. Bone resorption pits were stained with toluidine blue to evaluate bone resorption activity of osteoclasts. Besides, RAW264.7 cells were incubated in 96-well osteo assay surface plates (Corning Osteo Assay Surface) at a density of 1 × 10^4^ cells/well and induced with RANKL (100 ng/ml) and M-CSF (50 ng/ml) with or without IFN-λ1 for 5 days. Cells were removed with a 10% sodium hypochlorite and washed for three times with distilled water. The bone resorption pits were observed and photographed by light microscope. The absorption area was analyzed using the Image J software (NIH, Bethesda, MD). Other details were described in a previous report^12^.

### Confocal observation of NF-κB p65 and NFATc1 nuclei translocation

The cells were fixed with 4% paraformaldehyde for 20 minutes, then pass through the permeation and blocking procedure before being incubated overnight with the primary antibody at 4 °C. Cells were washed three times with PBS, followed by Alexa Fluor 488-conjugated secondary antibody and DAPI staining in dark condition. P65 protein and nuclei were observed by confocal fluorescence microscopy (Leica, TCS-SP5, DM6000-CFS). As for observation the nuclei translocation of NFATc1 after adding IFN-λ1, RAW264.7 cells were incubated with RANKL for 24 h in the presence or absence of 100 ng/ml IFN-λ1. Additional procedures were as same as previous descried. Gray value was elevated by using Image J software (the National Institutes of Health, USA).

### RNA isolation and quantitative real-time PCR (qRT-PCR) analysi**s**

Total RNA was extracted from different groups of osteoclasts by using TRIzol reagent (Life Technologies). Single-stranded complementary DNA (cDNA) was prepared from 1 μg of total RNA using reverse transcriptase with oligo-dT primer according the manufacturer’s instructions (Promega, USA). Two microlitres of each cDNA was subjected to PCR amplification. Then, real-time quantitative PCR (qPCR) was performed to investigate the expression of the indicated genes using SYBR Premix Ex Taq II (Takara Bio) in a PCR detection system (Bio-Rad, Hercules, CA, USA). Primer sequences are as shown in Supplementary Table 1. GAPDH were used as internal control.

### Western blot analysis

Cells were lysed in ice-cold RIPA lysis buffer (Beyotime, China) containing protease inhibitors, incubated on ice for 20 min, and centrifuged at 4 °C at 12,000 × *g*. For western blots, 30 μg of protein samples were subjected to sodium dodecyl sulfate–polyacrylamide gel electrophoresis followed by transfer onto PVDF membranes. After blocking in 5% skim milk, membranes were incubated with rabbit antibodies against primary antibodies overnight at 4 °C followed by 1 h-incubation with secondary antibody (1:2000). Blots against β-actin served as loading control. For densitometric analysis, Image J software (the National Institutes of Health, USA) was used.

### Calvarial osteolysis mouse model, micro-computed tomography (µCT) analysis, and histological analysis

Twenty-four healthy 8-week-old C57/BL6 female mice were obtained from the animal center of Third Military Medical University. The whole procedure was performed according to the guidelines of the animal care and use committee of Third Military Medical University. The mice were randomly divided to three groups: sham (injection with PBS), LPS (LPS treatment with 10 mg/kg), IFN-λ1 treatment (LPS treatment and injection with 0.2 mg/kg IFN-λ1). The detailed methods of subcutaneous injections were described previously^24^. All animals were anesthetized by an intraperitoneal injection of 4% chloral hydrate (5 ml/g) to reduce the level of suffering. The heads of the anesthetized mice were shaved to receive subperiosteal injections of PBS, LPS, or LPS + IFN-λ1. The total volume of the injections was 100 ml each time. Subsequently, the injections were administered every alternate day over a 14-day period.

For µCT analysis, a Bruker micro-CT Skyscan 1272 system (Kontich, Belgium) with an isotropic voxel size of 8.0 μm was used to image the whole calvaria. The scans were obtained in 4% paraformaldehyde using an X-ray tube potential of 60 kV, an X-ray intensity of 166 μA, and an exposure time of 1700 ms. The threshold for the calvaria bones was set at 86–255 (8-bit gray scale bitmap). Micro-CT scans of whole bodies of mice were obtained using an isotropic Voxel size of 148 μm. Reconstruction was accomplished using NRecon (Ver. 1.6.10). Realistic 3D-Visualization software (Bruker-micro-CT, Konitch, Belgium) was used to reconstruct the µCT images in three-dimension, which were acquired as ~2000 cross-sections. Three-dimensional and two-dimensional analyses were performed using CT Analyzer software (Ver. 1.15.4.0). All images presented are representative of their respective groups.

For the histological analysis of the bone tissue, the calvarias were dissected and fixed in 4% paraformaldehyde for 48 h. The calvarias were then decalcified by daily changes of a 15% tetrasodium EDTA soaking solution for 2 weeks at 37 °C. The decalcified calvarias were dehydrated by passing them through a series of increasing concentrations of ethanol, cleared in xylene twice, embedded in paraffin, and sectioned into 5 μm thick sections. The decalcified sections were completed with immunochemistry staining, hematoxylin and eosin (H&E) staining and masson staining according to the previous methods. The data obtained were used to describe the inflammatory response and osteoclast formation in vivo.

### Murine serum collection and ELISA assay

For detecting the inflammatory cytokines in blood of above mouse model, serum IL-1β, IL-6, and TNF-α levels were detected using mouse IL-1β, IL-6, and TNF-α ELISA kit according to the manufacturer’s protocol. The serum of mice was obtained from whole blood after standing for 30 min at room temperature and centrifugation at 3000 × *g* for 10 min at 4 °C. The absorbency of each standard and sample was measured at 450 nm. Standard concentration gradient was used as a standard curve.

### Data and statistical analysis

Statistical analyses were performed using GraphPad Prism 7 (GraphPad Software, La Jolla, CA). All data are presented as mean ± standard deviation (SD). Comparisons were analyzed using the unpaired, two tailed Student’s *t*-test and one-way analysis of variance (ANOVA), followed by the Bonferroni post hoc test to determine the significance of differences between two groups. Value of *p < 0.05, **p < 0.01, and ***p < 0.001 were considered as statistically significant.

## Supplementary information

Supplementary figure legends

Supplementary Table 1

Supplementary Figure 1

Supplementary Figure 2

## References

[1] Waldvogel FA (1970). Osteomyelitis: a review of clinical features, therapeutic considerations and unusual aspects. N. Engl. J. Med..

[2] van Staa TP (2006). Clinical assessment of the long-term risk of fracture in patients with rheumatoid arthritis. Arthritis Rheum..

[3] Gravallese EM (1998). Identification of cell types responsible for bone resorption in rheumatoid arthritis and juvenile rheumatoid arthritis. Am. J. Pathol..

[4] Lew DP, Waldvogel FA (2004). Osteomyelitis. Lancet.

[5] Cassat JE (2013). A secreted bacterial protease tailors the Staphylococcus aureus virulence repertoire to modulate bone remodeling during osteomyelitis. Cell Host Microbe.

[6] Gupta A (2014). Long-term outcome of pyogenic vertebral osteomyelitis: a cohort study of 260 patients. Open. Forum Infect. Dis..

[7] Gabriel M (2017). Inflammatory osteolysis: a conspiracy against bone. J. Clin. Invest..

[8] Wojdasiewicz P (2014). The role of inflammatory and anti-inflammatory cytokines in the pathogenesis of osteoarthritis. Mediators. Inflamm..

[9] Wang Y (2017). Staphylococcal protein A promotes osteoclastogenesis through MAPK signaling during bone infection. J. Cell. Physiol..

[10] O’ Brien T (1982). Acute haematogenous osteomyelitis. J. Bone Jt. Surg. Br..

[11] Teitelbaum SL (2000). Bone resorption by osteoclasts. Science.

[12] Chen Y (2018). Inhibitory effect of vanillin on RANKL-induced osteoclast formation and function through activating mitochondrial-dependent apoptosis signaling pathway. Life Sci..

[13] Zhang L (2020). Sphingosine-1-phosphate (S1P) receptors: promising drug targets for treating bone-related diseases. J. Cell. Mol. Med..

[14] Chen Y (2016). Alliin Attenuated RANKL-induced osteoclastogenesis by scavenging reactive oxygen species through inhibiting Nox1. *Int*. J. Mol. Sci..

[15] Takayanagi H (2002). RANKL maintains bone homeostasis through c-Fos-dependent induction of interferon-[beta]. Nature.

[16] Takayanagi H (2002). Signaling crosstalk between RANKL and interferons in osteoclast differentiation. Arthritis Res..

[17] Anson KA (2009). Mechanisms of interferon-β effects on bone homeostasis. Biochemical. Pharmacol..

[18] Kotenko SV (2011). IFN-lambdas. Curr. Opin. Immunol..

[19] Kotenko SV (2003). IFN-lambdas mediate antiviral protection through a distinct class II cytokine receptor complex. Nat. Immunol..

[20] Osterlund PI (2007). IFN regulatory factor family members differentially regulate the expression of type III IFN (IFN-lambda) genes. J. Immunol..

[21] Megjugorac NJ (2009). Modulation of human plasmacytoid DC function by IFN-lambda1 (IL-29). J. Leukoc. Biol..

[22] Xu L (2015). Interleukin-29 induces receptor activator of NF-κB ligand expression in fibroblast-like synoviocytes via MAPK signaling pathways.. Int. J. Rheum. Dis..

[23] Tang R (2019). Interleukin-37 inhibits osteoclastogenesis and alleviates inflammatory bone destruction. J. Cell. Physiol..

[24] Yang J (2019). Diallyl disulfide alleviates inflammatory osteolysis by suppressing osteoclastogenesis via NF-kB-NFATc1 signal pathway. Faseb. J..

[25] Asagiri M (2005). Autoamplification of NFATc1 expression determines its essential role in bone homeostasis. J. Exp. Med..

[26] Chen K (2019). Pseurotin A inhibits osteoclastogenesis and prevents ovariectomized-induced bone loss by suppressing reactive oxygen species. Theranostics.

[27] Ishii T (2018). OC-STAMP promotes osteoclast fusion for pathogenic bone resorption in periodontitis via up-regulation of permissive fusogen CD9. Faseb. J..

[28] Alippe Y (2017). Bone matrix components activate the NLRP3 inflammasome and promote osteoclast differentiation. Sci. Rep..

[29] Qu C (2015). NLRP3 mediates osteolysis through inflammation-dependent and -independent mechanisms. Faseb. J..

[30] Swanson, K. V. et al. The NLRP3 inflammasome: molecular activation and regulation to therapeutics. *Nat. Rev. Immunol*. **29**, 10.1038/s41577-019-0165-0 (2019). 10.1038/s41577-019-0165-0 PMC7807242 31036962

[31] Yu R (2019). Inhibition of HMGB1 improves necrotizing enterocolitis by inhibiting NLRP3 via TLR4 and NF-κB signaling pathways. J. Cell. Physiol..

[32] Zhou Z (2008). HMGB1 regulates RANKL-induced osteoclastogenesis in a manner dependent on RAGE. J. Bone Miner. Res..

[33] Xu J (2000). Cloning, sequencing, and functional characterization of the rat homologue of receptor activator of NF-kappaB ligand. J. Bone Miner. Res..

[34] Wang C (2003). 12-O-tetradecanoyl phorbol-13-acetate (TPA) inhibits osteoclastogenesis by suppressing RANKL-induced NF-kappaB activation. J. Bone Miner. Res..

[35] Ghosh S, Karin M (2002). Missing pieces in the NF-kappaB puzzle. Cell.

[36] Yamashita T (2007). NF-kappaB p50 and p52 regulate receptor activator of NF-kappaB ligand (RANKL) and tumor necrosis factor-induced osteoclast precursor differentiation by activating c-Fos and NFATc1. J. Biol. Chem..

[37] Pauli EK (2008). Influenza A virus inhibits type I IFN signaling via NF-kappaB-dependent induction of SOCS-3 expression. PLoS. Pathog..

[38] Li J (2013). JAK-STAT and bone metabolism. Jak. Stat..

[39] Lv N (2009). JANEX-1, a JAK3 inhibitor, protects pancreatic islets from cytokine toxicity through downregulation of NF-kappaB activation and the JAK/STAT pathway. Exp. Cell. Res..

[40] Takayanagi H (2009). Osteoimmunology and the effects of the immune system on bone. Nat. Rev. Rheumatol..

[41] Krishnamurthy A (2019). Citrullination controls dendritic cell transdifferentiation into osteoclasts. J. Immunol..

[42] Lapérine O (2016). Dendritic-cell-derived osteoclasts: a new game changer in bone-resorption-associated diseases. Drug. Discov. Today.

[43] Siegel R (2011). Regulation of IFN-λ1 promoter activity (IFN-λ1/IL-29) in human airway epithelial cells. J. Immunol..

[44] Hammad H, Lambrecht BN (2008). Dendritic cells and epithelial cells: linking innate and adaptive immunity in asthma. Nat. Rev. Immunol..

[45] Alnaeeli M (2006). Immune interactions with CD4 + T cells promote the development of functional osteoclasts from murine CD11c + dendritic cells. J. Immunol..

[46] Lazear HM (2015). Interferon-λ: immune functions at barrier surfaces and beyond. Immunity.

[47] Plotkin LI (2019). RAGE signaling in skeletal biology. Curr. Osteoporos. Rep..

[48] Sheikh F (2014). An essential role for IFN-β in the induction of IFN-stimulated gene expression by LPS in macrophages. J. Leukoc. Biol..

[49] Seeliger C (2015). Signaling pathway STAT1 is strongly activated by IFN-β in the pathogenesis of osteoporosis. *Eur*. J. Med. Res..

