# Supplementary material for: Dendritic cells-derived interferon-λ1 ameliorated inflammatory bone destruction through inhibiting osteoclastogenesis
Source: Cell Death Dis. 2020 Jun 2;11(6):414. doi: 10.1038/s41419-020-2612-z (PMC7265503; doi:10.1038/s41419-020-2612-z)
Supplement: Supplementary file 1 — Supplementary figure legends [file 41419_2020_2612_MOESM1_ESM.docx]

**Supplementary Figure Legends**

**Supplementary Table 1: Primer Sequences for qPCR.**

**Supplementary Figure 1. LPS could not make cytotoxicity.** (A) CCK-8 was performed in triplicate to analyze the cell viability of RAW264.7 cells treated with varying doses of LPS for 24h. (B) CCK-8 was performed in triplicate to analyze the cell viability of RAW264.7 cells treated with varying doses of LPS for 72h. The data in the ﬁgures represent the averages ±SD. N.S. represented as no significant difference. Signiﬁcant differences are analyzed using Student’s t test unless otherwise speciﬁed.

**Supplementary Figure 2. IFN-λ1 could inhibit LPS-induced osteoclastogenesis.** (A) Representative TRAP stain images of RAW264.7 cells treated with DMEM, LPS (100ng/mL), pre-treatment with RANKL for 24h and DMEM for another 48h, pre-treatment with RANKL for 24h and LPS for another 48h, pre-treatment with RANKL for 24h and LPS in presence of IFN-λ1 for another 48h. Scale bar=200μm. (B) Quantiﬁcation of osteoclasts number per well. The data in the ﬁgures represent the averages ±SD. N.S. represented as no significant difference. Signiﬁcant differences are indicated as *p<0.05 or **p<0.01 paired using Student’s t test unless otherwise speciﬁed.
