# Supplementary material for: Dendritic cells-derived interferon-λ1 ameliorated inflammatory bone destruction through inhibiting osteoclastogenesis
Source: Cell Death Dis. 2020 Jun 2;11(6):414. doi: 10.1038/s41419-020-2612-z (PMC7265503; doi:10.1038/s41419-020-2612-z)
Supplement: Supplementary file 2 — Supplementary Table 1 [file 41419_2020_2612_MOESM2_ESM.docx]

| **Genes** | **Forward** | **Reverse** |
| --- | --- | --- |
| PU.1 | 5ʹ‐GATGGAGAAGCTGATGGCTTGG‐3ʹ | 5ʹ‐TTCTTCACCTCGCCTGTCTTGC‐3ʹ |
| CTR | 5`-CGCATCCGCTTGAATGTG-3` | 5`-TC TGTCTTTCCCCAGGAAATGA-3` |
| CD9 | 5'-CGGTCAAAGGAGGTAG-3' | 5'-GGAGCCATAGTCCAATA-3' |
| mitf | 5'-AACTCCTGTCCAGCCAACCTTC-3' | 5'-TCTGCCTCTCTTTAGCCAATGC-3' |
| OC-STAMP | 5'-GGGCTACTGGCATTGCTCTTAGT-3' | 5'-CCAGAACCTTATATGAGGCGTCA-3' |
| CTSK | 5`-GAAGAAGACTCACCAGAAGCAG-3` | 5`-TCCAGGTTATGGGCAGAGATT-3` |
| c-Fos | 5`-CGGGTTTCAACGCCGACTA-3` | 5`-TTGGCACTAGAGACGGACAGA-3` |
| NFATc1 | 5`-CCCGTCACATTCTGGTCCAT-3` | 5`-CAAGTAACCGTGTAGCTGCACAA-3` |
| HMGB1 | 5`-TATCTAAATACGGATTGCTCAGGAA-3` | 5`-AGGGACAAACCACAATATAGGAAAA-3` |
| NLRP3 | 5`-GATCTTCGCTGCGATCAACAG-3` | 5`-CGTGCATTATCTGAACCCCAC-3` |
| TNF-α | 5`-AGGCGGTGCTTGTTCCTCA-3` | 5`-AGGCGAGAAGATGATCTGACTGCC-3` |
| IL-1β | 5`-CTCAACTGTGAAATGCCACC-3` | 5`-TGTCCTCATCCTGGAAGGT-3` |
| IL-6 | 5`-TGGGAAATCGTGGAAATGAGA-3` | 5`-ACTCTGGCTTTGTCTTTCTTGT-3` |
| β-actin | 5`-TCCCTGTATGCCTCTG-3` | 5`- ATGTCACGCACGATTT-3` |

**Supplementary Table 1:**
