# Supplementary figures and images for: Dendritic cells-derived interferon-λ1 ameliorated inflammatory bone destruction through inhibiting osteoclastogenesis
Source: Cell Death Dis. 2020 Jun 2;11(6):414. doi: 10.1038/s41419-020-2612-z (PMC7265503; doi:10.1038/s41419-020-2612-z)

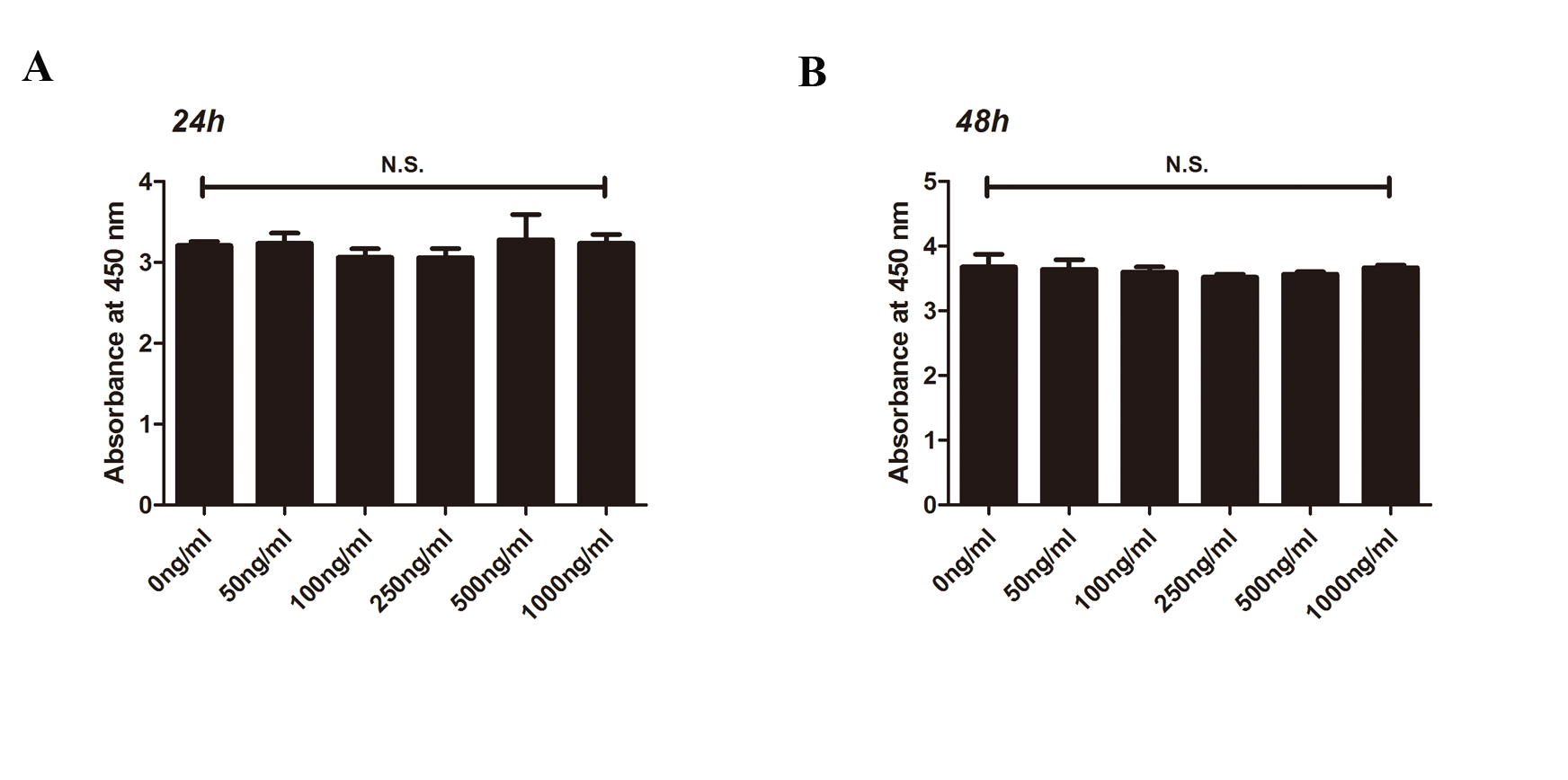

Supplement: Supplementary file 3 — Supplementary Figure 1 [file 41419_2020_2612_MOESM3_ESM.tif]

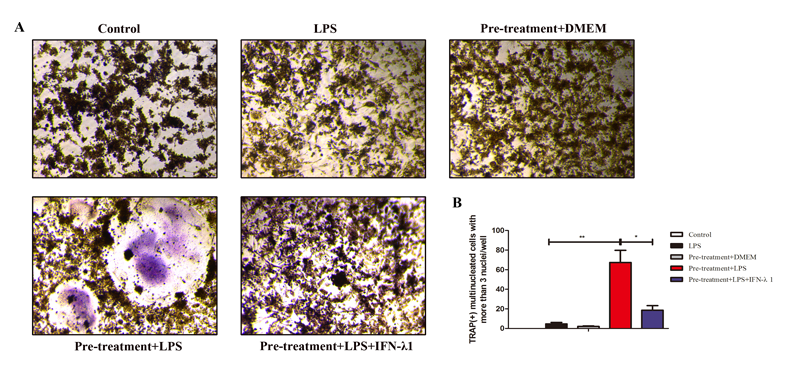

Supplement: Supplementary file 4 — Supplementary Figure 2 [file 41419_2020_2612_MOESM4_ESM.tif]
